# Teledentistry accuracy for caries diagnosis: a systematic review of in-vivo studies using extra-oral photography methods

**DOI:** 10.1186/s12903-024-04564-4

**Published:** 2024-07-22

**Authors:** Sanaz Kargozar, Mohammad-Pooyan Jadidfard

**Affiliations:** 1 Department of Community Oral Health, School of Dentistry, Shahid Beheshti University of Medical Sciences, Tehran, Iran; 2 Dental Research Center, Research Institute of Dental Sciences, Shahid Beheshti University of Medical Sciences, Tehran, Iran

**Keywords:** Dentistry, Oral health, Telemedicine, mHealth, eHealth, Dental caries, Dental decay, Diagnosis, Dental photography, Smartphone

## Abstract

**Background:**

Dental caries is a global public health concern, and early detection is essential. Traditional methods, particularly visual examination, face access and cost challenges. Teledentistry, as an emerging technology, offers the possibility to overcome such barriers, and it must be given high priority for assessment to optimize the performance of oral healthcare systems. The aim of this study was to systematically review the literature evaluating the diagnostic accuracy of teledentistry using photographs taken by Digital Single Lens Reflex (DSLR) and smartphone cameras against visual clinical examination in either primary or permanent dentition.

**Methods:**

The review followed PRISMA-DTA guidelines, and the PubMed, Scopus, and Embase databases were searched through December 2022. Original in-vivo studies comparing dental caries diagnosis via images taken by DSLR or smartphone cameras with clinical examination were included. The QUADAS-2 was used to assess the risk of bias and concerns regarding applicability. Meta-analysis was not performed due to heterogeneity among the studies. Therefore, the data were analyzed narratively by the research team.

**Results:**

In the 19 studies included, the sensitivity and specificity ranged from 48 to 98.3% and from 83 to 100%, respectively. The variability in performance was attributed to factors such as study design and diagnostic criteria. Specific tooth surfaces and lesion stages must be considered when interpreting outcomes. Using smartphones for dental photography was common due to the convenience and accessibility of these devices. The employment of mid-level dental providers for remote screening yielded comparable results to those of dentists. Potential bias in patient selection was indicated, suggesting a need for improvements in study design.

**Conclusion:**

The diagnostic accuracy of teledentistry for caries detection is comparable to that of traditional clinical examination. The findings establish teledentistry’s effectiveness, particularly in lower income settings or areas with access problems. While the results of this review is promising, conducting several more rigorous studies with well-designed methodologies can fully validate the diagnostic accuracy of teledentistry for dental caries to make oral health care provision more efficient and equitable.

**Registration:**

This study was registered with PROSPERO (CRD42023417437).

## Background

Dental caries is the most prevalent noncommunicable disease (NCD) and poses a significant public health challenge for populations and governments worldwide [1]. Untreated dental caries affects more than 2 billion people worldwide with permanent teeth and 514 million children globally with deciduous teeth. This condition ranks as the most widespread among all diseases in adults and is the most prevalent chronic childhood disease [2–4]. Untreated caries have various adverse effects across different phases of life and impose a substantial economic burden on society [1, 5]. If properly managed, dental caries is a preventable and reversible disease [6]. Early detection of lesions or individuals at high risk is of great importance in the prevention of caries [7].

Caries diagnosis involves the comprehensive assessment of available information, incorporating the identification and evaluation of caries signs (lesions) to determine the presence of the disease. The primary objective of caries diagnosis is to optimize patient health outcomes by choosing the most suitable management option for each type of lesion, providing information to the patient, and monitoring the clinical progression of the disease [8].

Numerous articles have reported only visual examination without the use of supplementary methods as the best strategy for caries diagnosis [9–13]. This approach established visual examination as the primary method for detecting caries lesions. However, obstacles such as travel and high costs can impose constraints, particularly when employing this method for population-based screenings or as a means of examining high-risk individuals living in remote and underserved areas [14]. Therefore, it would be of high priority to find cost-saving alternatives that can quickly detect caries and offer a diagnostic performance comparable to visual examination. Teledentistry, the remote diagnosis of dental diseases using transmitted photographic images of dentition, could serve as an alternative to visual inspection, especially for individuals living in remote or rural areas [15]. Synchronous (real-time) and asynchronous (store-and-forward) modalities are the most common forms of teledentistry [16]. In real-time modality, a live interaction between the health care provider and the patient, caregiver, or practitioner is established via audiovisual telecommunications technology. On the other hand, the store-and-forward modality involves the collection of health information at a specific time point, which is later shared with a practitioner [17]. The information transmitted between two sites can take various formats, such as data and text, audio, still images and video pictures [18].

Several studies have investigated the diagnostic performance of photographic methods for detecting dental caries. In a systematic review, Estai et al. evaluated the diagnostic accuracy of teledentistry in detecting caries compared to traditional nontelemedicine alternatives [21]. Moreover, Meurer et al. systematically reviewed the literature to determine the accuracy of dental images in diagnosing dental caries and enamel defects in children and adolescents [15]. According to the results of these reviews, the effectiveness of teledentistry in diagnosing common dental condition remains unclear, and the generalization of the results is difficult. Therefore, we decided to perform this systematic review to evaluate the diagnostic accuracy of teledentistry for detecting dental caries in permanent and primary dentition using photographs taken by DSLR and smartphone cameras.

## Methods

### Protocol and registration

The protocol of this systematic review was registered in PROSPERO (the International Prospective Register of Systematic Reviews, registration number CRD42023417437). To conduct the review process, the PRISMA-DTA reporting guideline (Preferred Reporting Items for Systematic review and Meta-analysis of Diagnostic Test Accuracy studies) was followed [22]. The PRISMA-DTA, an extension of the PRISMA statement, is designed to enhance the reporting quality of systematic reviews on diagnostic test accuracy (DTA) and to improve the comprehension of the performance of diagnostic tests.

### Eligibility criteria

According to the PRISMA-DTA reporting guideline, each component of the review questions should be detailed with respect to participants, index tests, and target conditions (PIT), which differs from the conventional PICO approach (participants, intervention, control, outcome) typically used in systematic reviews of intervention studies [22]. Therefore, to define the PIT components of our review question, we chose children and adults as participants; dental caries diagnosis via dental photographs taken by digital or smartphone cameras as the index test; and dental caries of primary and permanent dentition as the target condition. Visual or clinical examination of dental caries was considered the reference standard. As a result, studies were included if they compared the caries diagnosis of primary or permanent dentition from images (photographs taken by DSLR or a smartphone camera) as an index test to clinical examination as a reference standard in vivo setting.

Devices for capturing photographic images vary and include intra-oral devices, digital single-lens reflex (DSLR) cameras and smartphone cameras. Intraoral digital wand cameras are effective at capturing a single surface or one tooth in a single image. However the efficiency of these devices is under question when attempting to capture several teeth, a sextant or a quadrant in a single image [18]. DSLR and smartphone cameras are more attractive technologies due to their easy access and inherent imaging capabilities. The DSLR camera remains the most popular dental photography device [19]. A smartphone camera has proven to be easier to operate and handle than a DSLR camera. Furthermore, smartphones produce high-quality images compared with intraoral cameras [20].

Based on the above explanation and considering that intraoral cameras are expensive and often unavailable in remote and underserved areas, we excluded those studies that used intraoral cameras as a means of taking photographs for the teledentistry approach. Studies conducted on extracted teeth in vitro settings were also excluded.

We included original research studies in the review, including randomized clinical trials, quasi-experimental trials, longitudinal cohorts, and cross-sectional surveys. Case reports, position papers, reviews, and ongoing studies were excluded.

To optimize the sensitivity and eliminate potential bias, we did not consider any date restrictions when including studies.

### Information sources and search strategy

Electronic literature searches were conducted in February 2023 in the following databases: PubMed, Scopus and EMBASE. We also searched gray literature by combining words included in the search strategy using Google and Google Scholar. Our search was complemented by backward searching which is the process of manually searching the lists of references in identified publications or relevant reviews to identify any sources not accessible through systematic searches.

The search strategy employed a combination of medical subject headings (MeSH) and relevant text words within the field of study. The search procedures were customized for all databases, incorporating the appropriate syntax, subject headings, and controlled vocabulary to ensure the sensitivity of the search. No date restriction was used in the search strategies. We searched for studies in English or Persian language. The database search strategy was as follows:

(Telemedicine OR (Mobile AND Health) OR mHealth OR Telehealth OR eHealth OR e-medicine OR e-care OR ((Video OR Remote) AND Consultation*) OR Telecommunication* OR (telemedicine AND dentistry) OR teledentistry OR (dentistry AND (“intraoral photography” OR " dental photography “)) OR (telehealth AND dentistry) OR (Dental AND (“remote screening” OR teleconsultation OR telediagnosis))) AND (“dental caries” OR “tooth decay” OR “decayed teeth”) AND (“diagnostic accuracy”).

### Study selection

The identified articles from the search were transferred to Endnote 21 reference management software. A screening tool was developed according to the inclusion and exclusion criteria. One reviewer (SK) screened all the retrieved titles and abstracts first to remove duplicates and then for inclusion in the review according to the screening tool. The full texts of potential articles were obtained and evaluated to determine a study’s eligibility for inclusion in the full analysis. To avoid overlapping data, publications related to the same study were verified, and the most relevant report (according to study outcomes) was selected for full review.

### Data collection process

A data extraction form was developed to evaluate the selected articles. The form included the authors and year of publication, country, study setting, study design, sampling method, sample size, age range and sex of participants, reference standard, index test, type of outcome measure and main outcomes. One reviewer (SK) extracted the relevant data from the included full-text articles into an extraction form. The other reviewer (MPJ) independently checked and verified the extracted data. Any discrepancies between the two reviewers were resolved through discussion and consensus.

### Risk of bias and applicability

The quality assessment of the studies was assessed using the Quality Assessment of Diagnostic Accuracy Studies (QUADAS-2) tool established by Whiting et al. [23]. This instrument assesses the bias and applicability of included studies in relation to four distinct areas: patient selection, index test, reference standard and the flow and timing of patients within the study. The risk of bias was assessed in relation to the four domains. Applicability assessments were also conducted for the initial three domains. The applicability of evidence from a primary study is assessed in comparison to the review question. There was no overall summary score calculation; however, any concern regarding bias or applicability was categorized as ‘low’, ‘high’ or ‘unclear’ for each domain [24].

### Diagnostic accuracy measures

The main diagnostic accuracy measures to be extracted were sensitivity, which represents the likelihood of correctly identifying individuals with the disease, and specificity, which indicates the correct exclusion of disease in those without it. Additionally, we aimed to determine the positive and negative predictive values, which represent the probabilities that positive and negative test results correctly indicate or exclude disease, respectively, along with accuracy. Inter and intra-rater reliability were also assessed as additional outcomes.

### Synthesis of results

Due to substantial clinical, methodological, and statistical heterogeneity among the identified studies, the available data did not allow for a meaningful meta-analysis to be conducted. Meta-analysis is appropriate when a set of studies shows adequate homogeneity in terms of subjects, interventions, and outcomes, providing a meaningful summary to be generated [25]. Therefore, present review was not able to conduct a meta-analysis. We performed an extensive narrative synthesis of the data. The findings of the included studies are summarized and explained through textual descriptions and tables.

## Results

### Study selection

**Fig. 1:**
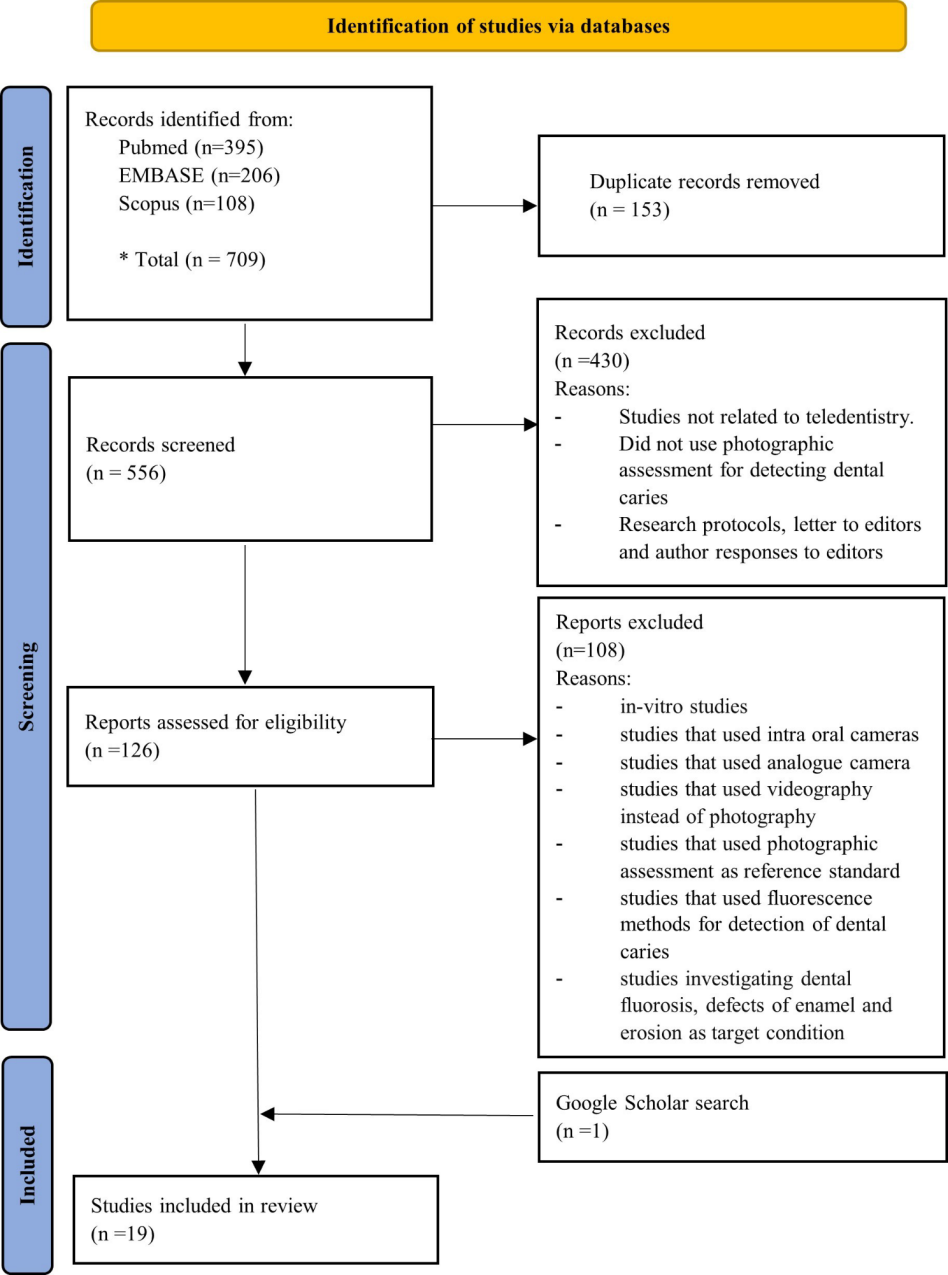
PRISMA flow diagram. (Adapted from PRISMA 2020 statement)

The electronic search identified a total of 709 studies from PubMed, EMBASE and Scopus. After removing duplicates, 556 studies remained for screening. Title and abstract screening resulted in the exclusion of 430 articles, the main reasons for which are described in the PRISMA flow diagram (Fig. 1). Among 126 potentially eligible studies, full-text screening excluded 108 studies that did not fulfill the inclusion criteria (Fig. 1). A search on Google Scholar led to the addition of one article to the included studies. The reasons for study exclusion are outlined in the subsequent flow diagram (Fig. 1). In the end, 19 studies were included in the final review.

### Study characteristics

The studies included in the analysis were conducted from 2014 to 2022, five of which were published in 2022. Altogether, five studies originated from Australia [14, 19, 26–28]; three from Saudi Arabia [29–31]; two from each of Brazil [32, 33]; China [34, 35]; and Iran [36, 37]; and one each from India [38], Sweden [39], the USA [40], Germany [7] and Italy [41].

The types of studies included observational cross-sectional studies [7, 19, 27, 28, 37], pilot intervention studies [26, 34, 41], retrospective descriptive studies [14, 42], and parallel-group randomized controlled trials [29]. Four of the studies were undertaken in schools [7, 19, 30, 35], one in a juvenile detention facility [33], and the remaining were carried out in dental clinics or hospitals [7, 14, 26–29, 31, 32, 34, 36, 38, 39, 41, 42]. The studies included participants with a wide age range from 1 to + 65 years. The majority of the reviewed studies (*n* = 11) did not explicitly report the sampling methods used. However, convenience sampling or voluntary participation was the most widely used sampling method. The sample sizes varied from 6 to 147 individuals, and 12 studies provided sample size estimates and power calculations.

To assess dental caries, various scoring systems were used, among which are the Decayed, Missing and Filled for primary and permanent teeth (dmft/DMFT) [7, 29, 30, 33, 34, 37], Decayed and Filled teeth (dft/DFT) [14, 19, 42], Decayed and Filling Surfaces (DFS) [40], International Caries Detection and Assessment System (ICDAS) [32, 39, 41] and ART caries assessment criteria [35]. Regarding the equipment type utilized for taking the photographs, 12 studies used a smartphone camera, one study [32] used both a smartphone and a conventional DSLR camera, and the remaining studies used DSLR cameras. All included studies used asynchronous or store and forward modalities of teledentistry. To send photographs for dental practitioners to diagnose dental caries at a distance, some studies used a type of data management software, e.g., Remote-I [14, 19, 26–28], and some used a kind of file sharing service [31, 33, 40]. Two studies used smartphone-based applications (e.g., WhatsApp) to send photographs to investigators [29, 38], and two studies used e-mails for this purpose [7, 35]. Mid-level dental providers/Oral hygiene therapists were responsible for remote caries assessments in 4 studies [14, 19, 27, 40]. In one study, children’s mothers performed caries diagnosis based on smartphone-based photographs [38]. Table 1 presents the methodological characteristics of the included studies.

**Table 1:** Study characteristic [methodological characteristic] of included studies (NM: not mentioned)

	Author (year) /Country	Total Sample Size Gender	Age Group	Caries Classification Index/Scoring System	Photograph Forwarding Method	Who performed photographic Assessment? Did the same person perform clinical examination?
1	Morosini et al. (2014) [33] / Brazil	- 102 Brazilian juvenile offenders - 100% male	15 to 19 years old [ mean age = 16.84 years (SD = 0.941)]	DMFT index	- In a file-sharing service (www.sendspace.com), and then the link was sent via e-mail to a distant consultant - On a compact disc	- Two other different distant examiners - No
2	Almosa et al. (2014) [39] / Sweden	- 89 patients treated with upper and lower fixed appliances and representing buccal caries lesions - NM	NM	A modified ICDAS-II scores	The photographs shown to the examiners in a random order	- Thirteen postgraduate orthodontics students with at least 2 years of experience as general practitioners - No
3	Estai et al. (2015) [26] / Australia	- 6 adult volunteer patients - Male (N=5) , female (N=6)	22 to 61 years old	Dental caries or existing restoration	Remote-I (a secure online server)	- Two independent offsite dental practitioners - No
4	Estai et al. (2016) [27] / Australia	- 100 participants - Male (64%), female (36%)	1 to + 65 years old	Sound or carious/root caries (filled and missing teeth were excluded)	Remote-I (a secure online server)	- Two Australian registered Mid-Level Dental Providers - No
5	Hu et al. (2016) [35] / China	- 115 sealed first molars - NM	NM	ART caries assessment criteria	The Word document of the photographs was sent to examiners.	- Two trained and calibrated examiners - Yes
6	Daniel et al. (2017) [40] / USA	- 78 children - Male (37%), female (63%)	4–7 years old	DFS index	Intraoral images posted on a specified site within Blackboard (a course management software)	- Two teledentistry examiners (dental hygienist and dentist) - No
7	Estai et al. (2017) [28] / Australia	- 100 participants - Male (64%), female (36%)	1 to + 65 years old	Sound or carious/root caries (filled and missing teeth were excluded)	Remote-I (a secure online server)	- Two off-site dentists (charter) - No
8	Kohara et al. (2018) [32] / Brazil	- 15 children (the occlusal surfaces of primary molars) - NM	3 to 6 years old	ICDAS scores	Images were randomly transferred to two computers	- Two experienced clinicians - Yes
9	Park et al. (2018) [14] / Australia	- 77 patients who underwent dental treatment under general anesthesia - NM	2 to 18 years old [mean age = 12.1 years (SD 3.5)]	All teeth were classified as either sound, carious, or restored	Remote-I (a secure online server)	- One MLDP (a registered dental therapist) - No
10	Kale et al. (2019) [38] / India	- 100 children and their mothers - NM	3–5 years old (4.1 ± 0.63)	Dental caries assessment criteria: the WHO 1997 criteria	WhatsApp	- Mothers - No
11	Alshaya et al. (2020) [31] / Saudi Arabia	- 57 children - Male (*N* = 32), female (*N* = 25)	6–12 years old [mean age = 7.79 years (SD ± 1.52)]	The WHO oral health assessment form for children (version 2013)	- An online cloud platform (Google Drive). - The sharing link was forwarded to the participating dentists using a social media application (WhatsApp Messenger, Facebook Corp., Mountain View, CA)	- Six pediatric dentists - No
12	Estai et al. (2021) [19] / Australia	- 138 children - 67 (48.6%) boys and 71 (51.4%) girls	a mean age of 7.8 ± 2.1 years old	dft/DFT index	Remote-I (a secure online server)	- Four trained OHTs - No
13	Guo et al. (2021) [34] / China	- 31 healthy college students - Women 48.4%, men 51.6%	18 to 35 years old [mean age, 19.29 years]	DMFT and DMFS indexes	Photos were stored on a computer for subsequent IDPE.	- Two trained dentists - Yes
14	Mehdipour et al. (2021) [37] / Iran	- 147 children - Girls 65.1%, boys 34.9%	11–12 years old [mean 11.68]	DMFT index	NM	- A dentist - Yes
15	Aboalshamat et al. (2022) [29] / Saudi Arabia	- 70 participants (35 in study group + 35 in control group) - female 68.6%, male 31.4%	mean age =32.3 ± 11.3 years old	DMFT index	WhatsApp	- NM exactly (the authors/research team) - NM
16	Alshaya et al. (2022) [30] / Saudi Arabia	- 95 children (Out of 120 eligible participants) - 54 (56.8%) boys and 41 (43.2%) girls	5–10 years old (a mean age of 7.8 ± 1.5 years)	dmft/DMFT index	Photographs were saved to a PC.	- A dentist - Yes
17	Golsanamloo et al. (2022) [36] / Iran	- 20 pediatric dental patients - 8 males and 12 females	between 6 to 12 years (mean age: 7.8 years)	The treatment plan for carious teeth according to the chart for registration of oral findings	NM	- 40 undergraduate dental students - Yes
18	Ciardo et al. (2022) [7] / Germany	- 50 patients - Female (80%), male (20%)	70.98 ± 7.60 years old	DMFT index + number of implants	The investigator’s own notebook or computer	- Clinical reference examiner and ten additional blinded raters (7 dentists + 3 dental students) - No
19	Zotti et al. (2022) [41] / Italy	- 43 patients (students of the Faculty of Dentistry) - 21 females and 22 males	between 22 and 38 years old (24.5 ± 2.7)	ICDAS II scores	- All the patients’ photos were received via a special email set up for this study. - Photos were stored and classified in the PC.	- An expert clinician - No

### Diagnostic outcomes

The most common diagnostic measures in the included studies, reported by 13 out of 19, were sensitivity and specificity. The sensitivity of photographic assessments for caries diagnosis varied between 48% and 98.3%, while the specificity ranged from 83 to 100%. In addition, 10 of these 13 studies reported positive and negative predictive values. For evaluating the diagnostic reliability of teledentistry in caries detection inter- and intra-examiner kappa statistics were used in 13 (with results ranging from 0.44 to 0.91) and 6 (0.52 to 1.00) studies, respectively. The mean d/D, dft/DFT or dmft/DMFT scores were calculated in 8 studies. In 6 studies, there were no significant differences in DMFT/DFT/DFS scores between the teledentistry assessment and clinical examination. In one study [14], the photographic assessment underestimated the dft/DFT scores, and the differences were more substantial in posterior tooth assessments than in anterior teeth assessment. One study [29] indicated that decayed teeth and total DMFT scores were significantly greater (overestimated) with teledentistry. The diagnostic outcomes of the included studies are presented in Table 2.

**Table 2:** Diagnostic outcomes of included studies

	Author (year)	sensitivity	specificity	PPV	NPV	accuracy	Inter examiner reliability (kappa statistic)	Intra examiner reliability (kappa statistic)	Mean d/D or dft/DFT or dmft/DMFT scores difference	Correlation: (Spearsmann correlation)
1	Morosini et al. (2014) [33]	48 - 71%	97 - 98%	85 - 89%	94 - 96%	93 - 95%	0.78- 0.86	-	-	-
2	Almosa et al. (2014) [39]	-	-	-	-	-	0.52–0.80	0.52–0.83	-	0.76
3	Estai et al. (2015) [26]	57%	100%	-	-	-	0.70	-	-	-
4	Estai et al. (2016) [27]	60 - 68%	97 - 98%	57 - 66%	97 - 98.5%	95 - 97%	0.57 - 0.61	0.89	-	-
5	Hu et al. [35]	-	-	-	-	-	0.65 - 0.70	-	-	-
6	Daniel et al. [40]	-	-	-	-	-	-	-	The teledentistry dentist’s DFS scores were higher than those of the other three examiners. No significant difference between the DFS scores of the clinical dentist and the teledentistry dental hygienist (*P* > 0.10).	0.99
7	Estai et al. [28]	60 - 63%	96 - 99%	52-79%	97-99%	94-97%	0.54–0.66	0.84	-	-
8	Kohara et al. (2018) [32]	- lower than 40% in the detection of initial and moderate caries - 75-100% in the detection of extensive caries lesions	higher than 83%	-	-	-	- lower than 0.66 for all devices and two examiners - Higher than 75% for sound surfaces and extensive caries lesions.	-	-	-
9	Park et al. (2018) [14]	61.5% [anterior: 67% posterior: 59%]	95% [anterior: 96% posterior: 94%]	79% [anterior: 76% posterior: 81%]	88% [anterior: 94% posterior: 84%]	-	0.62 [anterior: 0.67 posterior: 0.59]	-	The photographic assessment underestimated the d/D scores. The differences were more substantial in posterior teeth assessments, compared to anterior.	-
10	Kale et al. (2019) [38]	88.3%	98.3%	92%	97%	96%	0.87	100%	-	-
11	Alshaya et al. (2020) [31]	-	-	-	-	-	0.812	-	-	-
12	Estai et al. (2021) [19]	58 - 80%	98.7 - 99.9%.	81 - 96%	98.5 - 99.5%	-	0.72–0.87	0.65–0.82	The mean d/D scores for the visual dental examination remained higher than the photographic method but were not significantly different (*P* ≥ 0.07).	-
13	Guo et al. (2021) [34]	57.7% for DMFT 48.1% for DMFS	95.2% for DMFT 98.6% for DMFS	43.5% for DMFT 41.8% for DMFS	97.2% for DMFT 98.9% for DMFS	92.9% for DMFT 97.6% for DMFS	0.46 for DMFT index 0.44 for DMFS index	-	There were no significant differences in DMFT and DMFS indexes between CE and IDPE.	-
14	Mehdipour et al. (2021) [37]	79%	94%,	99%	31%	-	-	-	-	-
15	Aboalshamat et al. (2022) [29]	-	-	-	-	- 74.3% (good accuracy) with the number of missing teeth - 71.4% (good accuracy) with the number of filled teeth - 40% (moderate levels of accuracy) with the number of decayed teeth.	-	-	Decayed teeth and total DMFT scores were significantly higher (overestimated) with teledentistry.	-
16	Alshaya et al. (2022) [30]	- Primary teeth 95 - 98.3% - Permanent teeth 80.8 - 88.5%	- Primary teeth, 94.3 - 98.3% - Permanent teeth 94.1 - 96.1%	- Primary teeth: 95.2 - 96.6% -Permanent teeth, 87.5 - 92%	- Primary teeth: 91.7 - 97% - Permanent teeth: 90.6 - 94.2%		- Primary teeth: 0.89 – 0.91 - Permanent teeth: 0.76 - 0.85		Caries prevalence in children with primary teeth upon clinical dental examination was similar to teledentistry examination. For the permanent teeth, caries prevalence was also similar upon visual dental and teledentistry examination.	
17	Golsanamloo et al. (2022) [36]	84.2% (For virtual treatment plans)	92.9% (For virtual treatment plans)						No significant difference in the percentage of carious teeth between clinical and virtual examinations (*P* > 0.05). No significant difference in the treatment plans of students and the gold standard (*P* > 0.05).	
18	Ciardo et al. (2022) [7]	-	-	-	-	-	-	(pairwise Gwet’s AC1s) [for decayed teeth] 0.829-0.848 (almost perfect agreement)	-	-
19	Zotti et al. (2022) [41]	74	99.1	91.7	96.4					0.816

According to the conclusions drawn from the included studies, in 17 of those studies, the diagnostic accuracy of the photographic method was comparable to that of visual caries assessment.

### Risk of bias and applicability

**Fig. 2:**
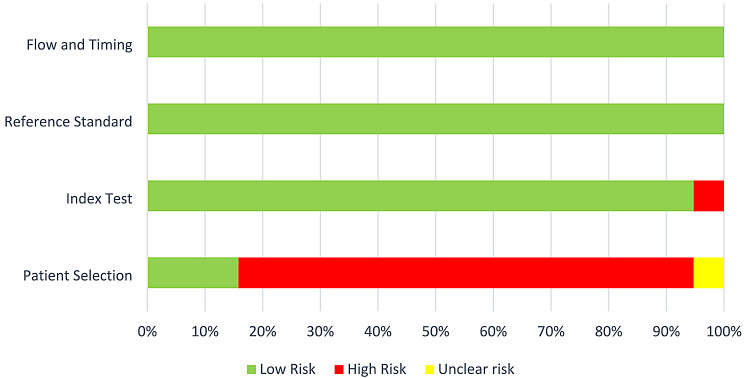
Percentage of articles with low, high, or unclear risk of bias

Table 3 displays the results of the quality assessment conducted for the 19 included studies. Bias occurs when systematic errors or limitations in the design or implementation of a study lead to distortions in the results [23]. The patient selection domain had the greatest impact on the risk of bias, being inadequate in 80% of the studies (Fig. 2). Convenience sampling was used in 6 studies. Only Hu et al. [35], Aboalshamat et al. [29] and Cardio et al. [7] used random sampling methods.

**Table 3:** Quality assessment outcomes of the selected studies using QUADAS-2 checklist

	Author (year)	Risk of bias				applicability		
		Patient selection	Index test	Reference standard	Flow and timing	Patient selection	Index test	Reference standard
1	Morosini et al. (2014) [33]	High	Low	Low	Low	Low	Low	Low
2	Almosa et al. (2014) [39]	High	Low	Low	Low	Low	Low	Low
3	Estai et al. (2015) [26]	High	Low	Low	Low	Low	Low	Low
4	Estai et al. (2016) [27]	Unclear	Low	Low	Low	Low	Low	Low
5	Hu et al. [35]	Low	Low	Low	Low	Low	Low	Low
6	Daniel et al. [40]	High	Low	Low	Low	Low	Low	Low
7	Estai et al. [28]	High	Low	Low	Low	Low	Low	Low
8	Kohara et al. (2018) [32]	High	Low	Low	Low	Low	Low	Low
9	Park et al. (2018) [14]	High	Low	Low	Low	Low	Low	Low
10	Kale et al. (2019) [38]	High	Low	Low	Low	Low	Low	Low
11	Alshaya et al. (2020) [31]	High	Low	Low	Low	Low	Low	Low
12	Estai et al. (2021) [19]	High	Low	Low	Low	Low	Low	Low
13	Guo et al. (2021) [34]	High	Low	Low	Low	Low	Low	Low
14	Mehdipour et al. (2021) [37]	High	Low	Low	Low	Low	Low	Low
15	Aboalshamat et al. (2022) [29]	Low	Low	Low	Low	Unclear	Unclear	Low
16	Alshaya et al. (2022) [30]	High	Low	Low	Low	Low	Low	Low
17	Golsanamloo et al. (2022) [36]	High	Low	Low	Low	Low	Low	Low
18	Ciardo et al. (2022) [7]	Low	High	Low	Low	Low	Low	Low
19	Zotti et al. (2022) [41]	High	Low	Low	Low	Low	Low	Low

**Fig. 3:**
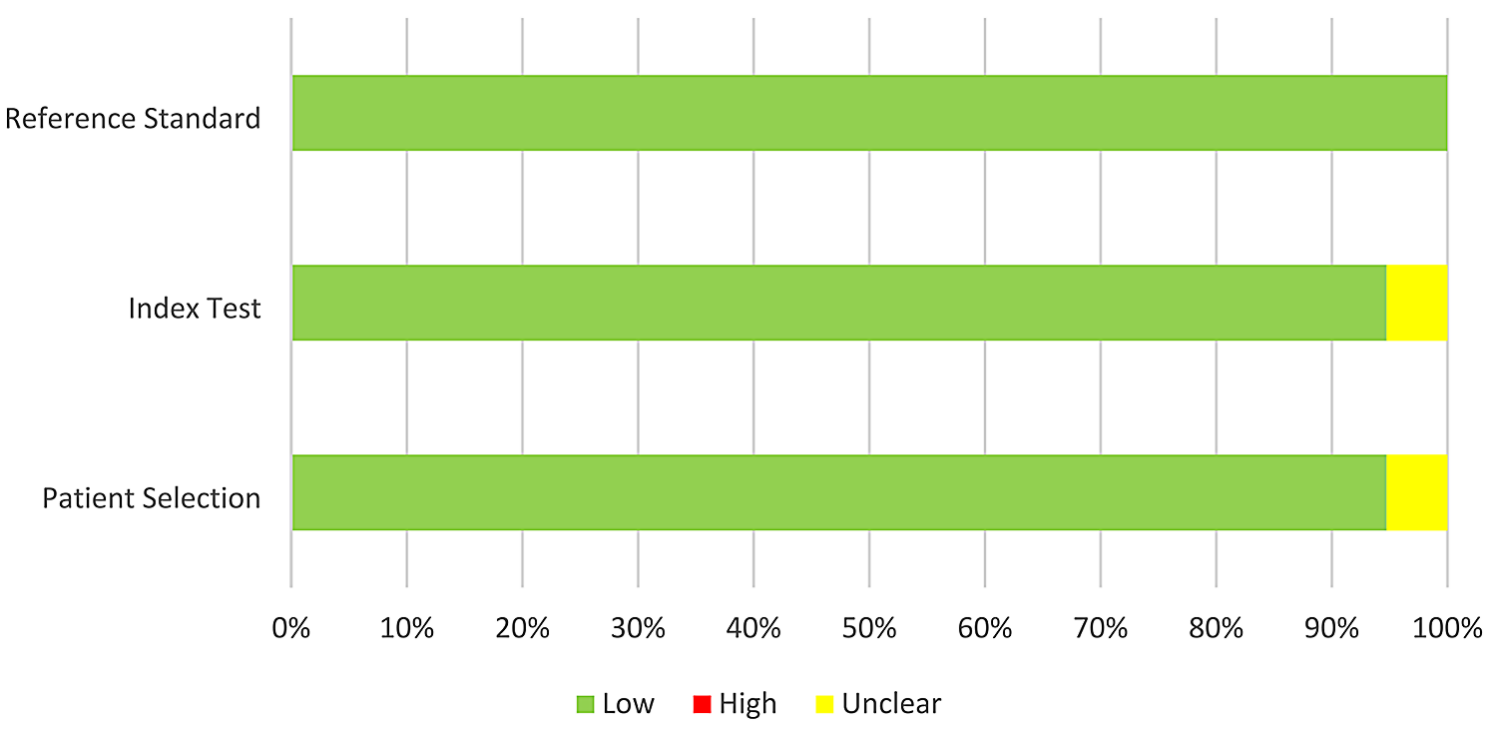
Percentage of articles with low, high, or unclear concerns regarding applicability

In 12 studies, the interpretation of index test results was performed without awareness of the results of the reference standard since practitioners who conducted the reference standard and the practitioners who interpreted the index test were not the same. Nevertheless, in those studies in which the reference standard and index test examiners were the same individuals, the authors considered a wash-out period that varied from two weeks [29, 30, 37] to one month [34], 45 days [32], or 10 months [35]. One study did not mention the wash-out period for one clinical examiner [7]. The reference standard and flow and timing domains were considered adequate in all studies (Fig. 2). All the included studies, except one [29], had good applicability (Fig. 3). A meta-analysis could not be conducted due to the variations in study design.

## Discussion

The primary finding of our systematic review was that a majority of the included studies (17 out of 19) indicated comparable diagnostic accuracy between the photographic method and visual assessment. The diagnostic outcomes reported in the included studies shed light on the effectiveness of teledentistry in the diagnosis of dental caries.

Sensitivity and specificity emerged as pivotal measures, illustrating the test’s ability to correctly identify positive and negative cases. The sensitivity, indicating the correct identification of evidence of caries formation, ranged from 48 to 98.3%, while the specificity, representing the ability to rule out caries or identify noncarious surfaces, ranged from 83 to 100%. Variations in performance were observed, attributable to factors such as study design, sample size, and chosen diagnostic criteria. For instance, in one study, the cutoff point adopted for calculating sensitivity and specificity was based on the presence or absence of untreated caries, with filled and missing teeth excluded from the analysis [33]. This definition was chosen because untreated caries has a more significant impact on both the patient and the healthcare system. Due to variations in the criteria, it is challenging to compare the sensitivity and specificity values of various studies. As a result, the heterogeneity observed among the identified studies prevents the conduction of a meaningful meta-analysis of the results.

Moreover, considering specific tooth surfaces and lesion stages is important when interpreting diagnostic outcomes. Three of the included studies investigated the feasibility of teledentistry for detecting dental caries across all stages of the disease [32, 39, 41]. For this purpose, they used the ICDAS as a clinical scoring system that allows the detection and assessment of caries activity. Kohara et al. [32] demonstrated that using smartphone images for photographic diagnosis is a viable and accurate approach for distinguishing between sound tooth surfaces and extensive caries lesions. However, this approach is not effective for accurately detecting initial and moderate lesions. In another study [39], the authors utilized images of the buccal aspects of teeth from patients who had recently undergone orthodontic treatment, and these images depicted caries lesions at various stages of progression. They concluded that evaluating buccal caries lesions on digital photographs based on the ICDAS-II is a reliable and valid method for assessing the severity of such lesions. According to Zotti et al. [41], telediagnosis of caries was found to be less sensitive than clinical diagnosis for detecting early-stage enamel caries, as the appearance of the lesion has not changed significantly at this stage.

Park et al. reported that photographic caries assessment provides an acceptable level of diagnostic detection, especially for anterior teeth, whereas this method led to underestimation of caries scores in posterior teeth assessments [14]. The difficulty in detecting carious lesions in posterior teeth from photographs may be due to confounding factors such as saliva, food debris, blood, and dental anatomy, particularly in the posterior region, which is less easily visible than in the anterior region. Another study [29] noted the potential for overestimation of decayed teeth and total DMFT scores through teledentistry. The authors of this article claimed that this overestimation of decayed teeth may be due to the presence of dental stains on the occlusal surface, which can be challenging to differentiate from occlusal caries, particularly when using low-quality photographs. Moreover, a six-month period between the clinical examination and the teledentistry session is sufficient for caries progression.

Among other diagnostic outcome measures were positive and negative predictive values, reported in 10 out of 19 studies, which underscored the clinical utility of teledentistry in predicting the presence or absence of dental caries. The reliability of diagnostic assessments through teledentistry was highlighted by the use of inter- and intra-examiner kappa statistics, with substantial agreement observed among the examiners.

The store-and-forward method was the method of choice in all 19 included studies. This method has demonstrated cost savings compared to real-time methods in dentistry and certain clinical disciplines [43–45]. However, this modality is not recommended for emergency cases [33].

Most of the included studies (12 out of 19) used smartphone-based dental photography. Owing to their digital photography capabilities and the computational power of smartphones, mobile devices are appealing technologies because they enable users to perform multiple tasks, such as processing, storage, and data transmission. In addition, smartphone cameras are readily accessible, lightweight, and very user friendly provide satisfactory images with minimal training [27]. While a DSLR camera excels in terms of flash units, illumination and image quality, and is capable of generating sharp images under low-light conditions or at high magnification, its relatively large size and weight reduce its convenience of use [20]. DSLR cameras also need a specific flash setup for optimal intraoral illumination, and their high costs might hinder their accessibility for dental providers in rural areas. An additional benefit of using a smartphone camera over a DSLR camera is that children are more accustomed to using a smartphone camera and are less intimidated than when using other photographic equipment [40]. The children’s natural affinity for smartphones and the enjoyable aspect of taking or having their pictures taken make the whole procedure more engaging and fun, fostering greater cooperation from the children [38]. In one of the studies included in our systematic review [32], two smartphones and a traditional macro-camera were used. The results of this study revealed no differences among the devices across all thresholds, and similar diagnostic performance was observed for the images captured with the three devices.

Remote screening for caries was accomplished by dentists or dental specialists in the majority of the studies. In 4 studies [14, 19, 27, 40] mid-level dental providers (MLDPs) were responsible for assessing intraoral photographs for dental caries. The results of these studies suggested that there is no significant difference between a dentist’s clinical identification of dental caries and the identification of dental caries by a MLDP based on photographs. Utilizing MLDPs provides a valid and reliable method for remote screening for caries.

It is worth noting that within our systematic review, one of the included studies [38] explored mothers’ capacity for diagnosing caries using the smartphone photographic method in comparison to a dentist’s clinical examination. The findings of this study suggested that providing dental health education to mothers about dental caries, its appearance, and associated signs and symptoms empowers them to diagnose their children’s dental caries with acceptable diagnostic accuracy.

It is clear that one crucial aspect in detecting dental caries with teledentistry is having a high-quality image of the dental arch, especially in posterior regions. In this regard, the existence of a specific protocol for capturing images and training individuals to take appropriate pictures is essential. Many of the reviewed articles in our systematic review involved a 20-minute training session for the designated photographer before initiating the patient’s image capture. In one of these studies, in which imaging was performed by a family member at home, a pre-written protocol was sent to the study participants [41]. Given the importance of image quality in teledentistry, developing a comprehensive and valid protocol for obtaining high-quality images using a digital camera or a mobile phone is recommended.

An evaluation of the risk of bias and applicability revealed insights into the methodological quality of the studies included. The patient selection domain posed the greatest risk of bias, with convenience sampling being utilized in a significant portion of the studies. Notably, only a few studies employed random sampling methods, indicating potential room for improvement in study design.

Regarding the interpretation of the results, most studies took precautions to ensure the independence of the index test examiners from the reference standard examiners. Wash-out periods were incorporated when the same examiner conducted both assessments. The reference standard and flow and timing domains were generally deemed adequate across studies, contributing to the overall reliability of the findings.

A notable distinction from prior studies, such as those conducted by Estai and Meurer, lies in our deliberate exclusion of investigations conducted in laboratory settings and those employing intraoral cameras. This decision was rooted in the recognition that intraoral cameras, while effective, are often costly and inaccessible to many dental facilities, especially in rural or remote areas. In contrast, digital and smartphone cameras are more widely available, with the added advantage of technological advancements that have significantly improved image quality.

The final review included 19 studies conducted between 2014 and 2022; the studies presented a diverse geographic distribution, had varying sample sizes, and included different age groups. Methodologically, the studies embraced a range of designs. This diversity offered a comprehensive perspective on the application of teledentistry in the diagnosis of dental caries across various settings and is in line with the aim of our systematic review being conclusive.

Nevertheless, this review has a number of limitations. Statistical heterogeneity among the studies identified did not permit to perform a meaningful meta-analysis of the results. However, we conducted a thorough narrative synthesis of the data, which allowed us to explore trends, patterns, and inconsistencies across studies. While narrative synthesis does not provide quantitative summary measures like meta-analysis, it still enables us to draw meaningful conclusions based on a qualitative analysis of the evidence. Our conclusions were based on the collective findings of the included studies, taking into account their strengths, limitations, and overall quality.

Single screening of the articles in the study selection phase is considered as another limitation of our study, although the selected studies have been randomly checked by another reviwer. Further, most of the studies were judged to have a high risk of bias in patient selection domain, which may reduce confidence in the findings.

## Conclusions

Although clinical examination remains the gold standard for diagnosing dental caries, current review shows a comparable diagnostic accuracy between teledentistry and traditional visual assessment across a wide variety of studies in terms of different factors, such as setting and sample size. These findings establish a solid foundation for the effectiveness of teledentistry, particularly in contexts with limited resources or difficulties accessing oral health services. Technological advancements, such as advancements in the quality of images captured using smartphone cameras or artificial intelligence (AI), promise an inevitable increase in the diagnostic accuracy of teledentistry systems in the near future. Taken together, the findings of this review, in conjunction with two prior reviews, can contribute to proving the accuracy of teledentistry in dental caries diagnosis, especially in remote and underserved areas. However, due to lack of clear or agreed-upon criteria to evaluate the conclusiveness of the findings of the systematic reviews, conducting a few more rigorous studies with well-designed methodologies can fully validate the diagnostic accuracy of teledentistry for dental caries to make oral health care provision more efficient and equitable.

## Data Availability

All data generated or analyzed during this study are included in this published article. All included articles in this review are available from SK on reasonable request.

## Abbreviations

MLDPs: Mid-Level Dental Providers
NM: Not Mentioned
